# Design and Verification of an Integrated Panoramic Sun Sensor atop a Small Spherical Satellite

**DOI:** 10.3390/s22218130

**Published:** 2022-10-24

**Authors:** Qi Zhang, Yulin Zhang

**Affiliations:** 1 College of Aerospace Science and Engineering, National University of Defense Technology, Changsha 410073, China; 2 College of Control Science and Engineering, Zhejiang University, Hangzhou 310000, China; 3 Huzhou Institute of Zhejiang University, Huzhou 313000, China

**Keywords:** Q-SAT, spherical satellite, attitude determination and control, IPSS, sun sensor

## Abstract

This paper proposes an integrated panoramic sun sensor (IPSS) for the small spherical satellite Q-SAT that has been working in orbit since 2020. IPSS is essentially a set of temperature-compensated photoelectric cells distributed on the spherical surface of Q-SAT. Compared with traditional sun sensors, IPSS has full spherical coverage of 4π so that the sun vector from any direction can be inversed. The mechatronic design and mathematical model of the proposed IPSS are presented. In-depth error analyses in terms of albedo effect, sampling error, parameter deviation, etc. are carried out. IPSS can provide a sun vector inversion accuracy of 1.5${}^{\circ }$ where albedo disturbance does not dominate. Simulation results show that the measurement of IPSS together with a COTS magnetometer can support the three-axis attitude determination of satellites in various orbits and can adapt to the seasonal variations of subpolar points. Ground experimental results and on-orbit data have also verified the feasibility and performance of IPSS. Although the panoramic sun sensor is designed for the small spherical Q-SAT, it can also be applied to other satellites with limited power budgets.

## 1. Introduction

This paper proposes the development and verification of an integrated panoramic sun sensor that can support satellite attitude determination with full spherical coverage. The background of the research is a spherical satellite called Q-SAT aimed at joint estimation of upper atmospheric density and long-wave gravity field in low-Earth orbit [1,2,3,4,5]. Q-SAT was launched atop the CZ-2D rocket on 6 August 2020, and has been working well for more than two years. The main payload of Q-SAT is a dual-frequency GNSS receiver, which provides cm-level orbit determination after postprocessing [3]. The precise orbit is used to inverse the aerodynamic drag force applied to Q-SAT, and thus the upper atmospheric density. The atmospheric density is related to the drag force as

(1) $$a_{drag}=\frac{1}{2}C_{D}\frac{A}{M}\rho V^{2}$$

The key parameter in the formula is the area-to-mass ratio $\frac{A}{M}$ in the windward direction. To minimize the error in this parameter, Q-SAT is designed to be spherical so that the area-to-mass ratio is constant under any attitude. The design of each subsystem of Q-SAT including the Attitude Determination and Control (ADC) subsystem must satisfy the goal of the spherical structure. Q-SAT has three axes stabilized to the Earth to keep the antenna pointing for telemetry and GNSS signal acquisition. The sun and geomagnetic vectors are used as the references for attitude determination to reduce the cost and power consumption (limited budget for body-mounted solar arrays) compared with more accurate star sensors.

There are various types of sun sensors, which differ in their technology and performance characteristics such as field of view (FOV), accuracy, power consumption, and size. The sun presence sensor is the simplest form that can only provide binary outputs and thus is insufficient for attitude determination. Digital sun sensors [6,7] have the highest accuracy up to about 0.01${}^{\circ }$. They usually achieve the measurement of sunray angles according to the spotted CCD images formed by sunlight illumination through masks of various shapes. However, digital sun sensors always suffer from the problem of a constrained field of view and relatively large power consumption due to their camera-like working principle. Typically, a digital sun sensor has a field of view up to 100${}^{\circ }$, and several sun sensors have to work concurrently to provide full spherical coverage [8]. The application of multiple sun sensors can further increase power consumption. Analog sun sensors [9] work by using the principle of the photoelectric effect and the cosine law of light intensity to calculate the sunray incidence. It requires at least 3 non-coplanar solar cells to determine the sun vector without uncertainty. When more valid solar cells are available, higher accuracy and stability can be achieved. Analog sun sensors are especially sensitive to albedo effects [10] and temperature variations. They are not as accurate as digital sun sensors. After correction, analog sun sensors can achieve an accuracy of up to 1.0–2.0${}^{\circ }$ when albedo reflections do not dominate. Although multiple solar cells are still required to provide full coverage, their power consumption is much smaller.

Considering the limited power budget and the less demanding task of the ADC system of Q-SAT, analog sun sensors are used. However, installing multiple commercial-off-the-shelf (COTS) sun sensors on the satellite surface will break the spherical structure. The COTS sensors can also take over a large part of the surface area that could have been used for mounting solar arrays. To address the problem, we developed IPSS to measure the sun vector in the satellite body frame. Extensive analyses, simulations, and experiments are carried out to verify the performance of the proposed IPSS. Q-SAT has 991 solar cells mounted on its spherical surface, among which 16 are used for the panoramic sun sensor. The 16 solar cells are evenly distributed so that at least 4 of them can be used under any attitude, providing enough redundancy and accuracy. Compared with COTS products, the panoramic sun sensor can be mounted in between the solar arrays and only occupies 0.78% of the surface area. To summarize, the proposed IPSS has the following advantages.

(1) IPSS has a panoramic field of view of 4π and can work under any attitude;
(2) When a subset of solar cells is damaged, IPSS can still provide reliable measurement;
(3) IPSS has a negligible power consumption;
(4) The spherical structure is maintained to the most compared with COTS products.

This paper focuses on the mechatronic design, mathematical modeling, error analysis, and verification of IPSS. Although IPSS is designed for the small spherical satellite Q-SAT, it can be applied to any satellite that has a limited power budget and does not require very high attitude determination accuracy.

The rest of this paper is organized as follows. In Section 2, the system overview, mechatronic design, and the sun vector inversion methods of IPSS will be introduced. In Section 3, we will give an in-depth error analysis of IPSS in terms of albedo effect, sampling error, parameter deviation, etc. Redundancy analyses are also presented in Section 3. Simulation and experiments that evaluate the overall design and the on-orbit performance of IPSS will be discussed in Section 4. Finally, we summarize in Section 5.

## 2. Mechatronic Design and Modeling of IPSS

In this section, an overview of the layout and working principle of Q-SAT will be introduced briefly. The mechatronic design as well as the sun vector inversion methods of the proposed IPSS will be presented in-depth.

### 2.1. Overview of the Small Spherical Satellite Q-SAT

**Figure 1:**
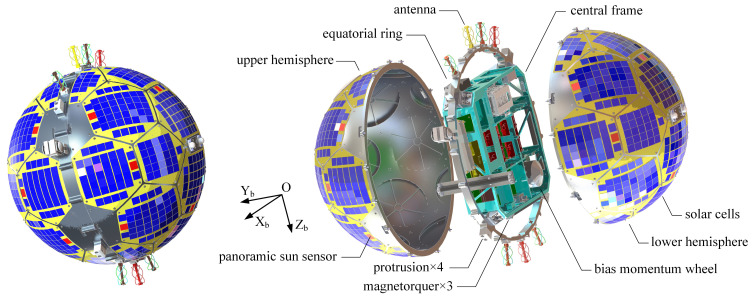
The structure, layout, and body frame definition of Q-SAT. Solar cells marked in red are used for joint estimation of the sun vector.

The structure, layout, and body frame definition of Q-SAT are shown in Figure 1. Q-SAT is a small spherical satellite with a diameter of 510 mm and weighs about 23 kg. The satellite consists of two hemispherical shells, an equatorial ring, and a cuboid central frame to install various onboard devices. The hemispherical shells are sculpted from single blocks of aluminum alloy to guarantee machining accuracy and overall strength. 20 pentagonal and hexagonal plates are installed on each hemispherical frame to seal the structure. Both hemispheres are attached to the equatorial ring, which also connects the cuboid central frame and the separation system. To guarantee the spherical structure, solar arrays are mounted on the surface of the two hemispheres. Q-SAT works in a 500 km sun-synchronous orbit. The specifications and orbit parameters of Q-SAT are summarized in Table 1.

The spherical Q-SAT was launched as a secondary payload, which is a challenging task. We have designed a customized electromagnetic separation system for Q-SAT to address the problem [4,5]. To provide interfaces with the separation system, 4 protrusions are designed around the equatorial ring of Q-SAT as shown in Figure 1. The separation system has two main functions: locking and release. The locking state is achieved by applying preloaded spring forces to the 4 protrusions which were also restricted by limit blocks of the separation system. In the locking state, Q-SAT is fixed to the separation system. Once the release signal is received, the separation system removes the limit blocks concurrently by a series of delicate transmissions using electromagnetic forces. When the limit blocks are drawn out, Q-SAT will be pushed into space by the preloaded spring forces. Q-SAT was released 60 s after the main payload.

Usually, Q-SAT keeps three axes stabilized to the Earth with axis $OZ_{b}$ pointing downward to the Earth center and axis $OX_{b}$ pointing forward. It uses three orthogonal magnetorquers and a bias momentum wheel installed in the body Y direction for active attitude control. The bias momentum wheel works in a constant speed mode to provide extra stability in inertial space to avoid attitude divergence under weak magnetic control. The sun vector and geomagnetic vector are used as references to determine the three-axis attitude of Q-SAT (only the geomagnetic vector is used when Q-SAT enters the shadow zone). The reference vectors can be obtained from the sun ephemeris [11] and the International Geomagnetic Reference Frame (IGRF) [12] with precise orbit data. The proposed IPSS and a COTS magnetometer are used for the sun and geomagnetic vector measurement in the body frame. As shown in Figure 1, solar cells marked in red are utilized for joint estimation of the sun vector.

### 2.2. The Integrated Panoramic Sun Sensor

**Figure 2:**
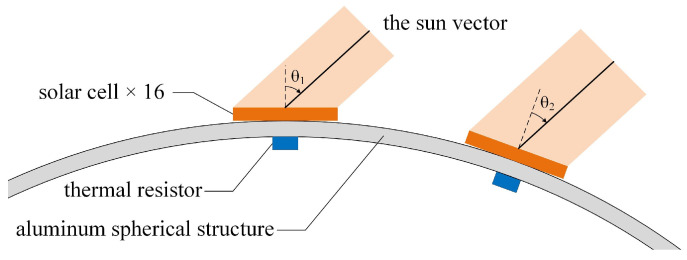
The IPSS consists of 16 evenly distributed solar cells, 16 thermistors underneath each solar cell, and corresponding sampling circuits.

As shown in Figure 2, the IPSS consists of 16 solar cells evenly distributed on the spherical surface, 16 thermal resistors for temperature compensation, and corresponding sampling circuits. The 16 solar cells are identical monocrystalline-silicon cells that are widely applied in solar power stations. The sun vector can be determined uniquely from at least 3 non-coplanar solar cells with valid measurements. IPSS is designed to have full spherical coverage of 4π and thus can provide immediate sunray vector estimation under any attitude. As to the other 975 cells for power supply, we use more energy-efficient triple junction GaAs cells for the limited space to install body-mounted solar arrays.

The size of the solar cells is customized (20 × 20 mm squares) to make the flat and rigid cells applicable to be mounted on the spherical surface. We have cut local flat platforms to ease the installation process of the 16 solar cells and to ensure the collinearity between their normal and the designed local radial directions. Thermal resistors are installed just beneath each solar cell to measure the time-varying temperature. The spherical structure is made of aluminum alloy (1.5 mm thick with stiffeners) with low heat resistance so that the measured temperature can reflect that of solar cells. The other 975 solar cells for power supply are directly mounted on the spherical surface without flat platforms.

1 Photoelectric Model of the Solar Cell

**Figure 3:**
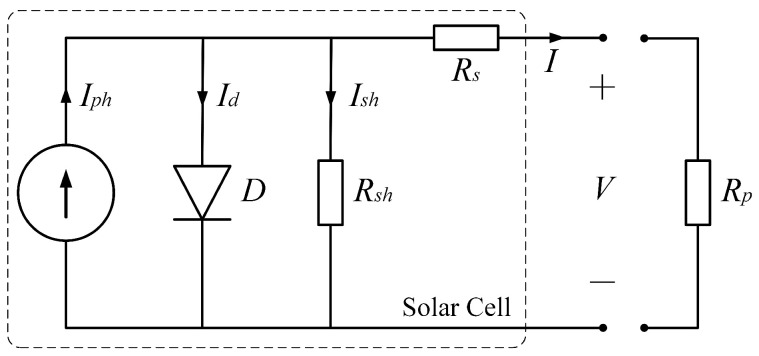
The equivalent circuit of a solar cell consists of a constant current source, a diode *D*, a shunt resistor $R_{sh}$, and a series resistor $R_{s}$. Resistor $R_{p}$ on the right-hand side is the payload resistor.

The electrical characteristics of the solar cell can be modeled as an equivalent circuit consisting of several ideal electronic components [13] as shown in Figure 3. According to the equivalent circuit, the volt-ampere relationship of a solar cell can be formulated as

(2) $$I=I_{ph}\left(E,\theta ,T\right)-I_{d}-I_{sh}=I_{ph}\left(E,\theta ,T\right)-I_{d,0}\left[exp\left(\frac{V+IR_{s}}{nV_{T}}\right)-1\right]-\frac{V+IR_{s}}{R_{sh}}$$

where *I* and *V* are the output current and voltage across the output terminals, $I_{ph}$, $I_{sh}$, and $I_{d}$ are the photogenerated current, shunt current and diode current respectively, $I_{d,0}$, $V_{T}$, and *n* are the reverse saturation current, thermal voltage and ideality factor of the diode (1 for the ideal diode) respectively, $R_{s}$ is the equivalent series resistance, and $R_{sh}$ is the shunt resistor.

Typically, the series resistance $R_{s}$ is magnitudes smaller than the shunt resistance $R_{sh}$. When the payload resistor $R_{p}$ is small enough, the output current *I* can be very close to the photogenerated current $I_{ph}$. In such a condition, the electric characteristic of a solar cell in Equation (2) can be simplified as a constant current source whose output is only related to the light intensity *E*, incident angle θ, and temperature *T*.

(3) $$I=I_{ph}\left(E,\theta ,T\right)$$

**Figure 4:**
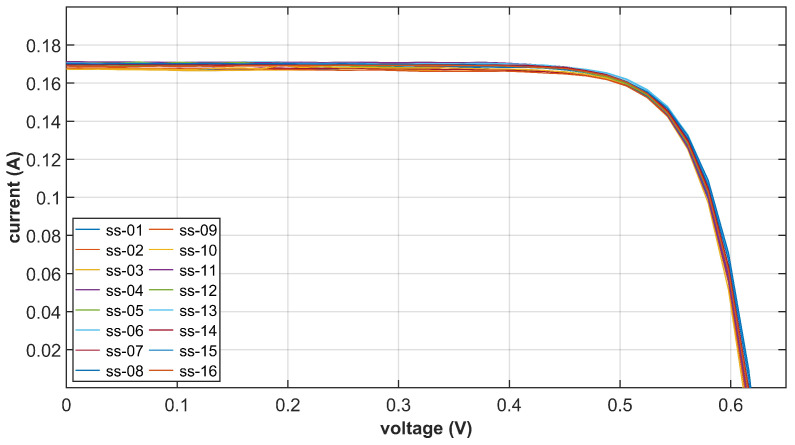
The volt-ampere curve of each solar cell under perpendicular illumination of the artificial sunlight. Solar cells with similar photoelectric characteristics are selected.

To validate this hypothesis, we tested the volt-ampere characteristic of each solar cell under artificial sunlight in a dark room. The artificial sunlight has the same light intensity and similar spectral distribution as the real sunlight in Earth’s orbit. Artificial sunlight is cast perpendicularly to the solar cell, and the exposure time is kept very short so that the temperature remains the same as the room temperature. Figure 4 gives the volt-ampere curve of all 16 selected solar cells to set up the IPSS. The output current of each solar cell is almost constant when the output voltage is below 0.45 V, which validates our hypothesis in Equation (3). It is also worth mentioning that the type of solar cells can also influence the result. We have selected solar cells that have strong constant current source characteristics.

2. The Kelly Cosine Characteristic of the Solar Cell

**Figure 5:**
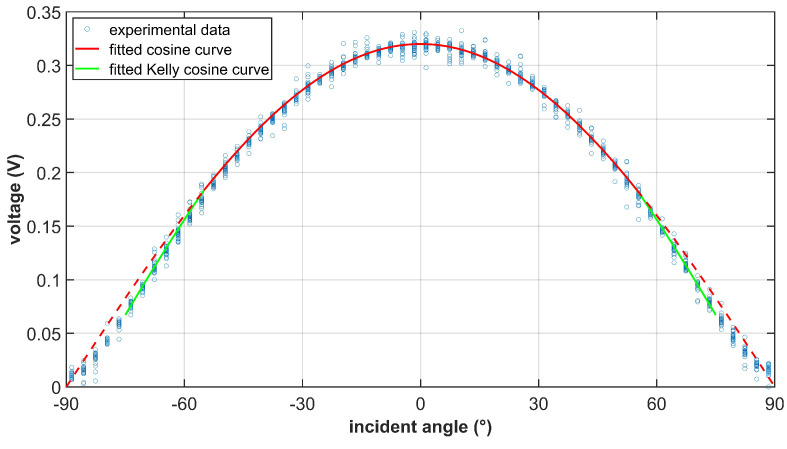
Sampled voltage curve of a solar cell with various incident angles. The proposed empirical formula fits the experimental data well. A 2−ohm precise resistor is connected in series to measure the current.

The sunlight intensity in low-Earth orbit can be assumed to be constant within a certain period of time (seasonal variation is about 3.4% due to the Earth’s elliptic orbit). Intuitively, the output current of the solar cell should conform to the cosine low when illuminated with different incident angles. However, the cosine relation is not exactly satisfied during ground experiments. In cases where the incident angle θ is large, the output current deviates and is slightly smaller than the reference cosine curve as shown in Figure 5. The experimental data satisfies the so-called Kelly cosine characteristic of photovoltaic cells [14].

We proposed an empirical formula to characterize the Kelly cosine relationship between the output current and the incident angle θ according to tremendous ground experimental data.

(4) $$I_{ph,T_{0}}=\max\left(I_{\max,T_{0}}\cos\left(\theta \right)-a\cdot u\left(\left|\theta \right|-\theta_{th}\right)\left(\left|\theta \right|-\theta_{th}\right),0\right)$$

where $I_{ph,T_{0}}$ is the photogenerated current at standard temperature $T_{0}$ (25 ${}^{\circ }$C), $I_{\max,T_{0}}$ is the reference photogenerated current when the sunlight is cast perpendicularly, $\theta_{th}$ is the incident angle threshold, *a* is a parameter that characterizes the magnitude of the deviation and is assumed to be a constant, u(·) is the step function. When the incident angle is larger than the threshold $\theta_{th}$, the photogenerated current gradually deviates from the standard cosine curve.

As shown in Figure 5, the proposed empirical formula fits the experimental data well, and is able to capture the Kelly cosine characteristic of the solar cell to be used. During the experiment, a 2-ohm precise resistor is connected in series to measure the current indirectly. The threshold angle $\theta_{th}$ is set to be 55${}^{\circ }$. When the incident angle is smaller than the threshold, the experimental data conforms to the standard cosine law. When the incident angle is larger than the threshold, the experimental data can be modeled by the empirical Formula (4) more precisely.

3. Temperature Correction

The photogenerated current of a solar cell is sensitive to variations in temperature. The effect of temperature is approximately linear [15,16] and must be considered to ensure the final accuracy of IPSS. The temperature compensation model of the output current is formulated as a linear function.

(5) $$I_{\max}=I_{\max,T_{0}}-K\left(T-T_{0}\right)$$

where $I_{\max}$ is the photogenerated current when the sunlight is cast perpendicularly, *K* is the temperature compensation coefficient, and *T* is the measured temperature of the solar cell.

To sum up, the output current of a solar cell is modeled as a function of the incident angle θ and temperature *T* as shown in Equation (6). With sampled current and temperature, the incident angle θ of the sunlight can be inversed with a simple calculation. During the calculation, measurements with small currents should be discarded since they can be easily disturbed.

(6) $$I_{ph}=\max\left(\left(I_{\max,T_{0}}-K\left(T-T_{0}\right)\right)\cos\left(\theta \right)-a\cdot u\left(\left|\theta \right|-\theta_{th}\right),0\right)$$

### 2.3. The Sun Vector Inversion Principle

In this subsection, we focus on the sun vector determination methods based on the proposed IPSS. The problem can be described as the estimation of the sun vector in the satellite body frame according to the measurements of *N* solar cells with known installation matrices. The observation model of the problem is straightforward, and can be formulated as

(7) $$\cos\left(\theta_{i}\right)=\langle {\vec{n}}_{i},\vec{s}\rangle ,i=1,2,\cdots ,N$$

where ${\vec{n}}_{i}$ is the installation vector of the $i_{th}$ solar cell, $\vec{s}$ is the sun vector to be estimated in the satellite body frame, $\theta_{i}$ is the sunlight incident angle of the $i_{th}$ solar cell that can be calculated using the empirical Kelly cosine Formula (4).

It is worth noting that not all measurements contribute the same to the final solution. As shown in Figure 5, the solar cell has low sensitivity when θ is small, and high sensitivity when θ is large. However, when θ is close to perpendicular, the measurement is more likely to be disturbed. Considering the above factors, we propose a weighted method to calculate the sun vector that can distinguish the contribution of each solar cell.

(8) $$\vec{s}={\left(A^{T}WA\right)}^{-1}WA^{T}\left[\begin{array}{c} \cos(\theta_{1})\\ \cos(\theta_{2})\\ \vdots \\ \cos(\theta_{N}) \end{array}\right]$$

where $A={\left[{\vec{n}}_{1},{\vec{n}}_{2},\cdots ,{\vec{n}}_{N}\right]}^{T}$ is the set of installation vectors, $W=diag(w_{1},w_{2},\cdots ,w_{N})$ is the corresponding weight matrix. The weight can be selected with different strategies, such as the square of the slop $\sin^{2}\theta$ or the simplest form as

(9) $$w_{i}=\left\{\begin{array}{ccc} 1 & , & \theta_{i}\leq \theta_{d}\\ 0 & , & \theta_{i}>\theta_{d} \end{array}\right.$$

The Kelly cosine curve is very close to that of the standard cosine curve when θ does not exceed the threshold $\theta_{th}$ too much. When calibrated parameters are not available, we can also inverse the sun vector by assuming the outputs conform to the standard cosine law and discard measurements with a large incident angle. Under such an assumption, Equation (8) can be further simplified as

(10) $$\vec{s}={\left(A^{T}WA\right)}^{-1}WA^{T}\left[\begin{array}{c} \frac{I_{ph,1}}{I_{\max,T_{0}}-K\left(T_{1}-T_{0}\right)}\\ \frac{I_{ph,2}}{I_{\max,T_{0}}-K\left(T_{2}-T_{0}\right)}\\ \vdots \\ \frac{I_{ph,N}}{I_{\max,T_{0}}-K\left(T_{N}-T_{0}\right)} \end{array}\right]$$

where $I_{ph,i}$ and $T_{i}$ are the sampled current and temperature of the $i_{th}$ solar cell respectively. We have selected solar cells with similar photoelectric characteristics, so the same parameter $I_{\max,T_{0}}$ is used. Although the second method in Equation (10) is a simplified version of Equation (8), it is more robust to the seasonal variations in sunlight intensity and does not require precise parameter calibration.

## 3. Accuracy and Redundancy Analyses of IPSS

### 3.1. Accuracy Analyses

The proposed IPSS is essentially a type of analog sun sensor. The performance of IPSS is affected by a variety of factors such as sampling error, parameter deviation, albedo effect, etc. A comprehensive list of factors is summarized in Table 2 and will be analyzed in depth individually.

1. Sampling Error

The output current and temperature are the two raw measurements that need to be sampled by the circuit. The magnitude of the sampling error directly affects the accuracy of the final solution. A 2-ohm precise resistor is connected in series to each solar cell to convert the output current to a voltage signal. Two 12-bit high-speed A/D converters with 8 channels are used to sample the voltage. The average of five consecutive samples is used to improve the sampling accuracy and stability.

**Figure 6:**
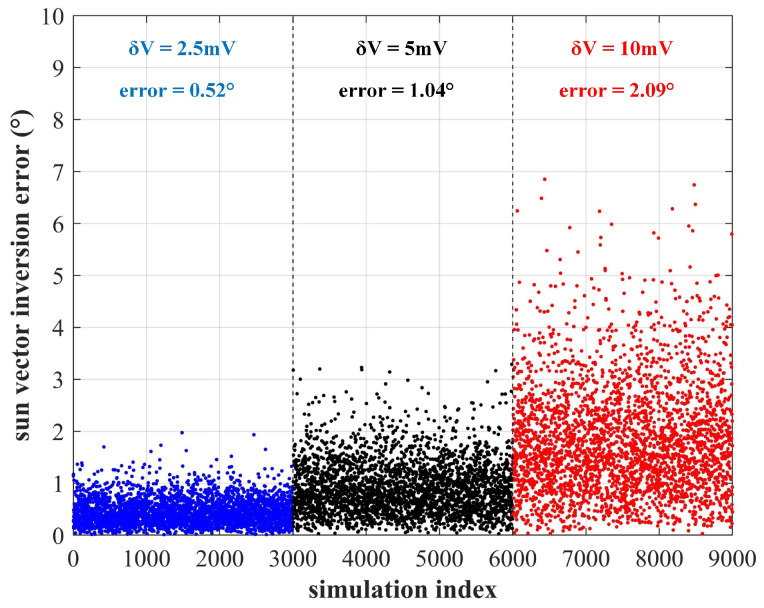
The sun vector inversion accuracy considering voltage sampling error of various magnitudes.

Experimental result shows that the average sampling accuracy is better than 5 mV, which is about 1% of the maximum voltage of the solar cells. Monte Carlo simulations were carried out to analyze the impact on the final sun vector solution. As shown in Figure 6, the average sun vector inversion accuracy is about 1.04${}^{\circ }$ considering a voltage sampling error of 5 mV. If a digital amplifier is used to amplify the voltage signal, the accuracy can be further improved.

In terms of temperature measurement, thermal resistors are mounted just beneath each solar cell for temperature correction. The spherical structure of Q-SAT is made of aluminum alloy (1.5 mm thick) with low heat resistance, and thus the measured temperature can reflect that of the solar cells. The temperature compensation coefficients of the solar cells are quite small (0.53 mA/${}^{\circ }$C). Even if we consider a large temperature sampling error up to 3 ${}^{\circ }$C, the simulated error of the inversed sun vector is only 0.64${}^{\circ }$, which is much smaller than that introduced by the current sampling error.

2. Manufacturing and Installation Error

The second type of error source is the manufacturing and installation error. The manufacturing error of the structure is quite small using a CNC machine and can be ignored. To minimize the installation error of the 16 flat solar cells on the spherical structure, we have cut local flat platforms to ease the installation process and to ensure the collinearity between the solar cells’ normal and the local radial direction. In such a condition, even if the installation position of the solar cells deviates, the installation matrix can still be close to the design value.

**Figure 7:**
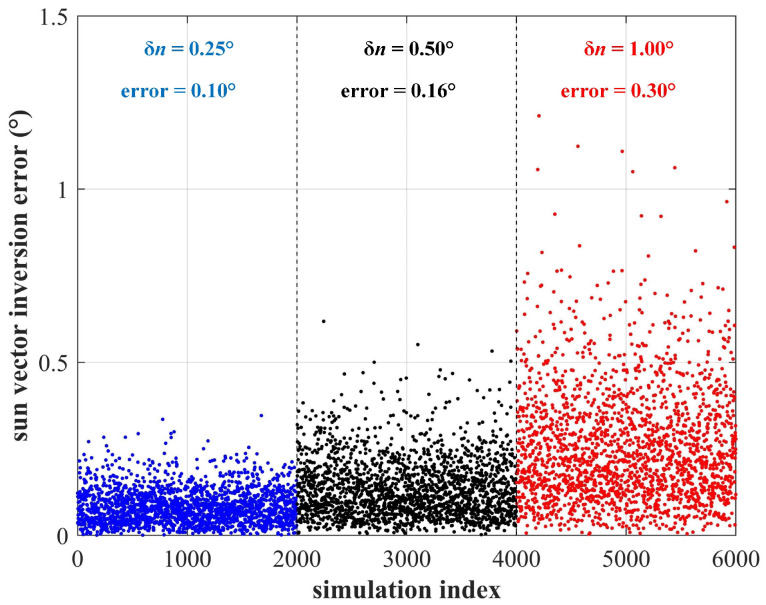
The sun vector inversion accuracy considering installation error of various magnitudes.

As shown in Figure 7, the installation error of solar cells has no significant impact on the final solution due to the averaging effect of multiple solar cells. In cases where the installation error reaches a large value of 0.5${}^{\circ }$, the sun vector inversion accuracy can still maintain 0.16${}^{\circ }$.

3. Parameter Calibration Error

A few parameters of IPSS need to be set or calibrated before launch, such as the resistance of the current sampling resistor $R_{p}$, the temperature compensation coefficient *K*, and the reference photogenerated current $I_{\max,T_{0}}$, etc. Their calibration error can also affect the accuracy of the inversed sun vector.

For the voltage sampling resistor, we have selected resistors with precise resistance that does not deviate more than 0.5% from their design value. Monte Carlo simulation shows that the resistance error can only introduce a sun vector inversion error up to 0.14${}^{\circ }$. The temperature compensation coefficient error can be calibrated below 10%. The error in *K* will have an equivalent impact as the voltage sampling error if only the solar cell’s temperature deviates 50 ${}^{\circ }$C from the standard temperature $T_{0}$. Normally, the deviation is much smaller according to the on-orbit data send back by Q-SAT.

**Figure 8:**
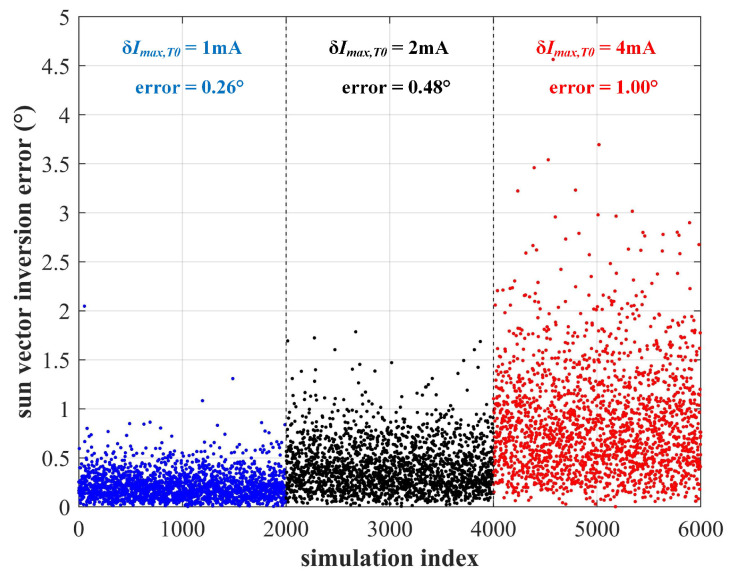
The sun vector inversion accuracy considering reference current error of various magnitudes.

In terms of the reference photogenerated current $I_{\max,T_{0}}$, the error cannot be greater than 2 mA if we set the value to the median of 169 mA as shown in Figure 4 (the constant current ranges from 167 mA to 171 mA). Figure 8 gives the Monte Carlo simulation result considering reference current error of various magnitudes. The error of 2 mA will introduce a sun vector inversion error of 0.52${}^{\circ }$. When the current error doubles, the sun vector inversion error reaches 1.07${}^{\circ }$. Therefore, it is quite important to select solar cells with similar photoelectric characteristics.

The results of the above error analyses have enabled us to focus on critical areas of IPSS to further improve its stability and accuracy.

4. Seasonal Variations in Sunlight Intensity and Earth Albedo Effect

The sunlight intensity has seasonal variations due to the elliptical orbit of the Earth around the sun. Considering the eccentricity (e = 0.0167) of the Earth’s orbit, the magnitude of variations can reach about 3.4% of the solar constant (or 46 W/m${}^{2}$). Solar activity may also introduce a variation, but much smaller than that caused by the change in the sun-Earth distance [17]. The sunlight intensity changes slowly around the year, and modified parameters can be uploaded to adapt to the variations. A more convenient and reliable approach is to use the second sun vector inversion method (10), which is by nature insensitive to the variation in light intensity. Mento Carlo simulation also shows that the seasonal variations can have a negligible impact on the final solution if the second sun vector inversion method is adopted.

**Figure 9:**
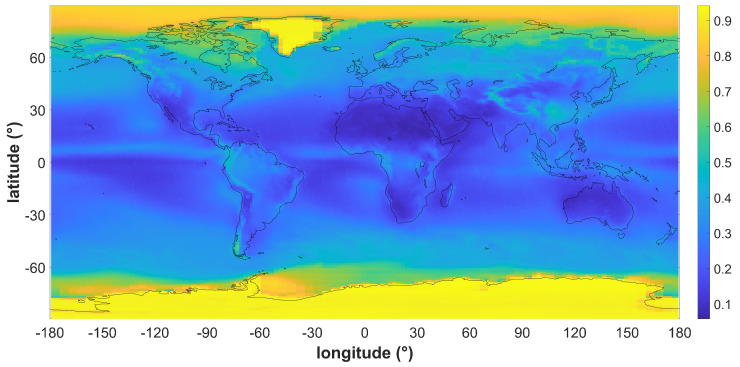
The average albedo coefficient measured by the TOMS−EP program.

The shorter-term light intensity variations in Earth’s orbit are caused by the albedo effect [10] or the reflectivity of the Earth. The intensity of reflected sunlight depends mostly on the ground surface property and the amount of cloud cover. The large area of ice cover and cloud cover are the primary determinants of global albedo. Figure 9 presents the albedo map collected by the Total Ozone Mapping Spectrometer Earth Probe (TOMS-EP) program [18]. The albedo coefficient can be up to 90% in ice-covered areas such as Antarctica and Greenland. It is also obvious from the map that the reflectivity has a large dependency on latitude, and only a small dependency on longitude.

**Figure 10:**
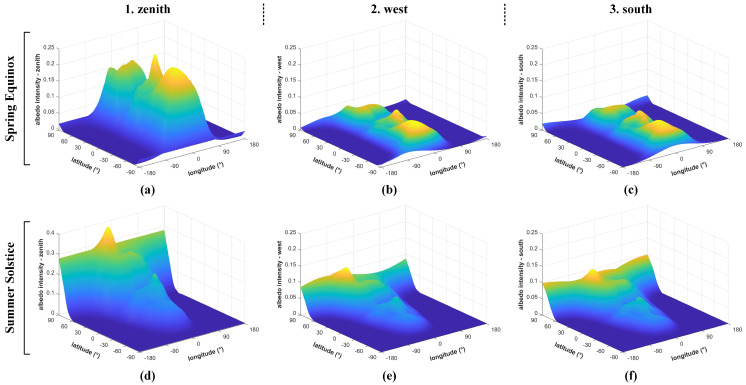
The albedo intensity in various directions normalized with respect to the solar constant at (**a**–**c**) the Spring Equinox and (**d**–**f**) the Summer Solstice at the altitude of 500 km.

**Figure 11:**
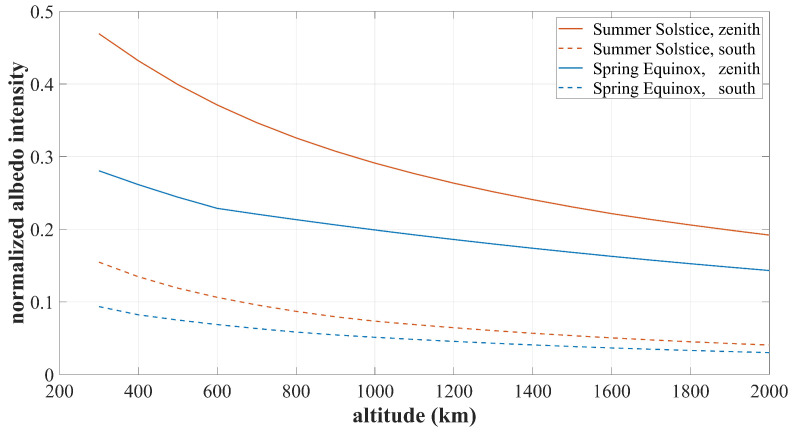
The albedo intensity normalized with respect to the solar constant at various altitudes.

The impact of the albedo effect on IPSS is complex. The influence can be significant or negligible depending on a variety of coupling factors such as the satellite’s orbit, the position of the subsolar point, and climate change. Q-SAT works in a 500 km sun-synchronous orbit. Figure 10 presents the albedo intensity distribution in various seasons and directions at an altitude of 500 km. The reflected sunlight has the highest intensity in the zenith direction and much smaller intensity in other directions. As shown in Figure 10a,d, the maximum albedo intensity at the Spring Equinox is about 25% of the solar constant, while that at the Summer Solstice can be up to 40% (about 540 W/m${}^{2}$). Figure 11 presents the change of the albedo intensity with respect to altitude. The albedo intensity drops dramatically with altitude and thus better performance of the proposed IPSS is expected for satellites in higher orbits. This paper mainly focuses on the 500 km orbit, which is of reference value to the applications of most small satellites.

The impact of the albedo effect can be mitigated in two ways. The first approach is to model and fit the albedo intensity, and try to remove the albedo components from the coupled measurements. However, the Earth’s albedo is not a simple parallel light with constant intensity but a complicated diffuse reflection from almost all directions. The time-variant climate change can also have a large influence which is hard to model. Moreover, the first approach can be computationally intensive and may not be suitable for real-time applications.

**Figure 12:**
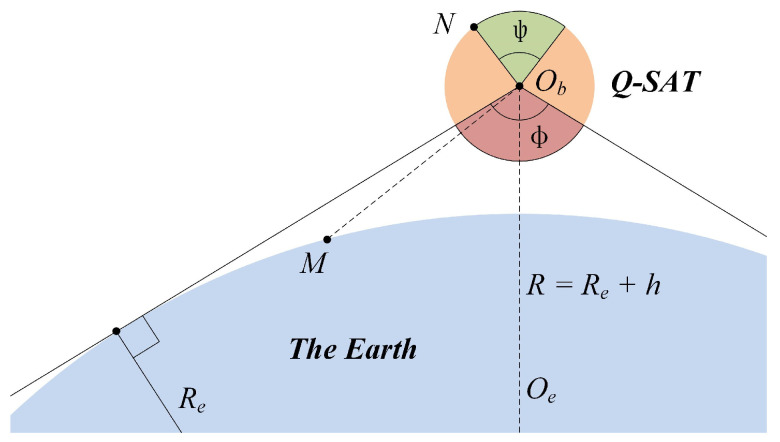
The impact of Earth albedo on different solar cells. $O_{b}M$ is the radius of the influence area of the albedo effect, and $O_{b}N$ is perpendicular to $O_{b}M$. Solar cells in red are sheltered from the sunlight. Solar cells in green cannot be influenced by the albedo effect. Solar cells in the yellow region can be influenced by both the sunlight and the albedo effect.

**Figure 13:**
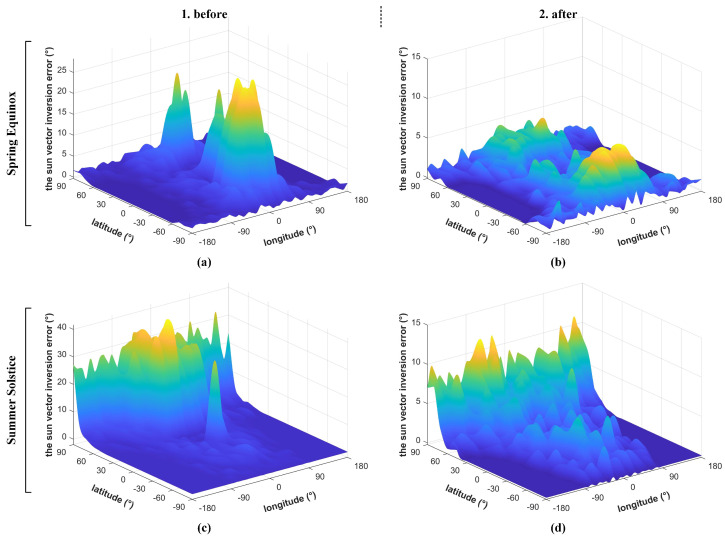
The sun vector inversion accuracy before and after invalid measurements culling. At the Spring Equinox (**a**,**b**) the overall accuracy improves from 4.84${}^{\circ }$ to 1.84${}^{\circ }$. At the Summer Solstice (**c**,**d**), the overall accuracy improves from 7.41${}^{\circ }$ to 2.16${}^{\circ }$.

The second approach does not try to decouple the components but discards solar cells (we have a total of 16 solar cells) with invalid measurements. As shown in Figure 12, the satellite surface marked in red (ϕ = 136${}^{\circ }$ at 500 km) is sheltered from the sun. Therefore, solar cells in the red region do not provide any valuable clues. On the contrary, the satellite surface marked in green (ψ > 44${}^{\circ }$ at 500 km) cannot be influenced by the albedo effect. Solar cells in the green region would provide the most valuable information. To mitigate the influence, we set a threshold of valid measurement according to the maximum possible reflection intensity (the threshold can have different values considering the orbit and seasons) so that solar cells in the red region can be completely discarded. When an initial attitude estimation is available, we can also discard the mostly corrupted measurements in the yellow region according to the sun vector and the nadir direction in the satellite body frame. As shown in Figure 13, by setting an appropriate threshold according to the maximum possible albedo reflection, the mean sun vector estimation error drops from the original 4.84${}^{\circ }$ to 1.84${}^{\circ }$ at the Spring Equinox and from 7.41${}^{\circ }$ to 2.16${}^{\circ }$ at the Summer Solstice.

The results in Figure 13 have considered all factors listed in Table 2 that may have an impact. The overall sun vector estimation accuracy is averaged better than 1.5${}^{\circ }$ where the albedo effect does not dominate. It is also worth mentioning that the overall error can be up to 10${}^{\circ }$ (mainly system error) when the albedo influence cannot be removed efficiently. These cases are rare and can be only found in high-latitude regions (to be aware that the Mercator projection has enlarged the area dramatically in polar regions) near Winter and the Summer Solstice. For Q-SAT, the task of the ADC system is to keep the antenna pointing for telemetry and GNSS signal acquisition. The simulation and on-orbit data in Section 4 show that the proposed IPSS is sufficient to fulfill the task.

### 3.2. Redundancy Analyses

The proposed IPSS not only has the merit of full spherical coverage, but also is designed to be fault tolerant with redundant measurements. When a small subset of solar cells is damaged, IPSS can still work with the rest measurements.

**Figure 14:**
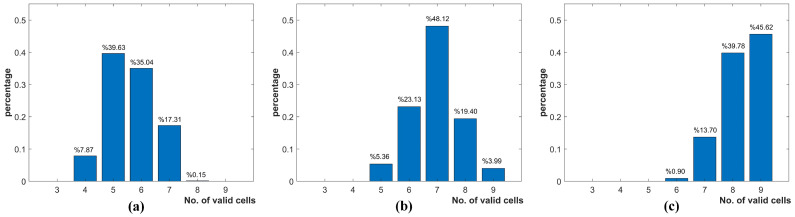
Percentage of solar cells with valid measurement when the threshold of (**a**) 65${}^{\circ }$, (**b**) 75${}^{\circ }$ and (**c**) 85${}^{\circ }$ are imposed respectively.

Figure 14 gives the number of available solar cells when different incident angle thresholds are imposed. As shown in Figure 14b, at least 5 solar cells can still provide valid measurements when a threshold of 75${}^{\circ }$ (measurements less than 26% of the solar constant are discarded) is imposed. Even though we set the threshold at the more critical 65${}^{\circ }$ (measurements less than 42% of the solar constant are discarded), the number of valid solar cells is still between 4 and 8.

## 4. Experimental Results and On-Orbit Performance

In this Section, ground experiments and simulations were carried out to evaluate the performance of the proposed IPSS. To be more convincing, we have also presented the on-orbit data to verify the overall design.

### 4.1. Ground Experiments with Artificial Sunlight

**Figure 15:**
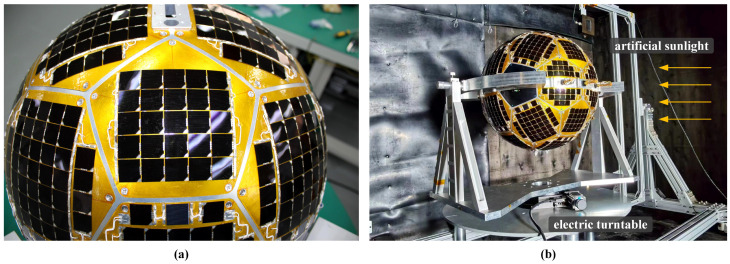
Q-SAT before launch. (**a**) Totally 991 solar cells are mounted on the spherical surface. (**b**) Q-SAT is undergoing the ground experiment to test the feasibility and performance of the proposed IPSS using artificial sunlight.

Artificial sunlight is used to test the performance of IPSS on the ground. As shown in Figure 15a, solar cells are mounted on the spherical surface of Q-SAT where 975 are used for charging and 16 are used to set up the IPSS. Figure 15b shows the scenario during the ground experiment. Q-SAT was placed within a large black box with the body Y axis pointing upward. The electric turntable downward Q-SAT can drive the satellite to rotate 360 degrees around its vertical axis with precise angular command. The artificial sunlight is cast towards the center of Q-SAT horizontally. The uniform area of the artificial sunlight is sufficient to cover the whole satellite. During the ground experiment, Q-SAT was rotated 30 degrees every 2 min, and the data was collected at 1 Hz.

**Figure 16:**
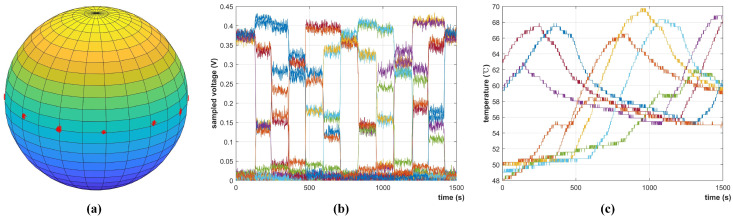
Raw measurements of IPSS and the inversed sun vector in satellite body frame during the ground experiment. (**a**) the inversed artificial sun vector in the satellite body frame. (**b**) sampled voltage curves of solar cells in the upper hemisphere. (**c**) sampled temperature curves of solar cells in the upper hemisphere.

Figure 16b,c give the sampled voltage and temperature of each solar cell during the ground experiment. As shown in Figure 16b, the sampled voltage is larger than that in Figure 5 since the artificial sunlight is not calibrated to but higher than the solar constant. Therefore, the experiment can also test the performance of IPSS with sunlight intensity variations. As is shown in Figure 16c, the temperature can be up to 70 ${}^{\circ }$C after long-time illumination. Figure 16a shows the sun vector inversion result where each red dot implies an inversed sun vector in the body frame. It can be seen that the distribution of the results has a small variation. IPSS is tested to have achieved an accuracy of 2.01${}^{\circ }$ compared with the reference angle provided by the electric turntable. The obtained accuracy is close to that considering the albedo effect as shown in Figure 13. The reflection and occlusion of the aluminum supporting structure have introduced the extra error, which resembles the albedo in low-Earth orbit to some extent. When we remove the system error, the average fluctuation of the inversed sun vector is around 1.5${}^{\circ }$.

### 4.2. Simulation in Various Orbits and Seasons

Simulations were carried out to verify the performance of IPSS to support three-axis attitude determination and control of satellites in various orbits and lighting conditions. We have considered several typical sun-synchronous orbits (local time of descending 12:00 and 18:00) and seasons (Spring Equinox and Summer Solstice). At the Summer Solstice, the subsolar point coincides with the Tropic of Cancer, and thus can have a larger influence on the IPSS when the satellite is in the Northern hemisphere.

During the simulation, IPSS and a simple magnetometer are used for attitude determination. A bias momentum wheel and 3 orthogonal magnetorquers are used for three-axis attitude control. The core parameters in the simulations are kept the same as those of Q-SAT, and are summarized in Table 3.

**Figure 17:**
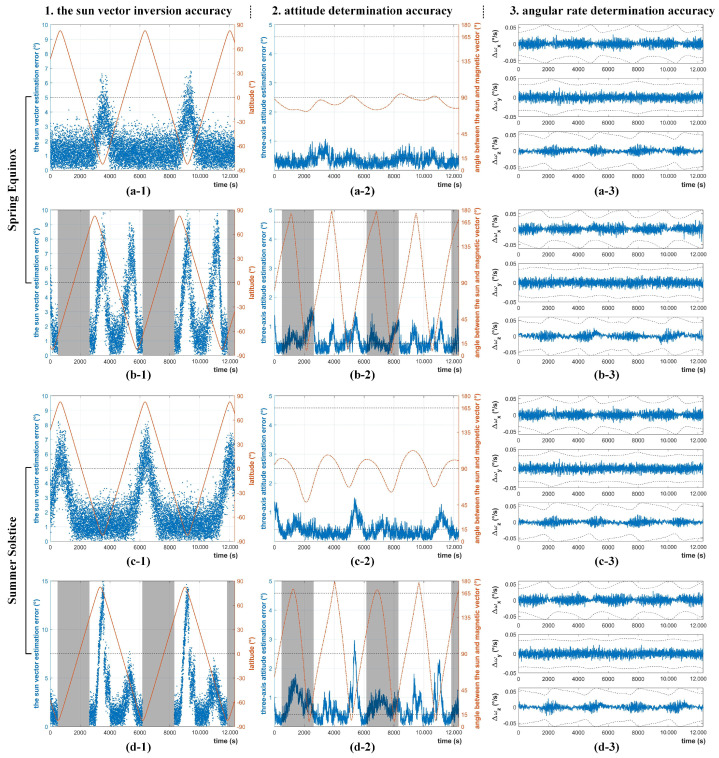
Performance of IPSS to support three-axis attitude determination in various orbits and seasons. (**a-1**,**a-2**,**a-3**) 18:00 sun−synchronous orbit at the Spring Equinox. (**b-1**,**b-2**,**b-3**) 12:00 sun−synchronous orbit at the Spring Equinox. (**c-1**,**c-2**,**c-3**) 18:00 sun−synchronous orbit at the Summer Solstice. (**d-1**,**d-2**,**d-3**) 12:00 sun−synchronous orbit at the Summer Solstice.

Figure 17a,b give the simulation results at the Spring Equinox when the subsolar point is exactly upon the equator. As shown in Figure 17a, the sun vector inversion error of IPSS in the dawn-dusk orbit (18:00) is averaged 1.62${}^{\circ }$. When the satellite is over the south polar region, the error goes up to about 5${}^{\circ }$ due to the strong albedo in Antarctica. The results are consistent with the error analyses shown in Figure 13. In the dawn-dusk orbit, IPSS together with a COTS magnetometer can achieve an average attitude determination accuracy of 0.32${}^{\circ }$ after filtering (such as a gyro-free MEKF filter [19]). As is shown in Figure 17b, the mean sun vector inversion error in the 12:00 orbit is 3.12${}^{\circ }$. The error can exceed 5${}^{\circ }$ at around 60${}^{\circ }$ N and 60${}^{\circ }$ S where the albedo reflection is coupled with the sunlight to the most. This is also consistent with the error analyses in Figure 13. The satellite can achieve an attitude determination accuracy of 0.43${}^{\circ }$ which is a bit larger than that of the 18:00 orbit (the attitude determination error can occasionally exceed 1${}^{\circ }$ when the sun and magnetic vector are near parallel). It is worth mentioning that the errors introduced by the albedo effect are mainly system errors that vary slowly. Therefore, the overall angular velocity estimation is quite stable and accurate, and can be better than 0.01${}^{\circ }$.

Figure 17c,d show the simulation results at the Summer Solstice when the subsolar point coincides with the Tropic of Cancer. As is shown in Figure 17c, the mean sun vector inversion accuracy in the dawn-dusk orbit is 2.30${}^{\circ }$. When the satellite is over the north polar region, the inversion error can go up to 7${}^{\circ }$ due to the strong albedo effect in polar regions. For the dawn-dusk orbit, an attitude determination accuracy of 0.38${}^{\circ }$ can be achieved after filtering. In terms of the 12:00 orbit, the average sun vector inversion accuracy in the southern hemisphere is improved, while that in the northern hemisphere is impaired since the change of subsolar point. As is shown in Figure 17d, the final attitude determination accuracy is not affected that much, and is averaged 0.61${}^{\circ }$ after filtering.

The overall performance of IPSS in the simulation is summarized in Table 4. The above simulation has verified the feasibility of the proposed IPSS. The measurement of IPSS can also support the attitude determination and control system of satellites working in various orbits and seasons. The average sun vector inversion accuracy of IPSS can be better than 1.5${}^{\circ }$ when the albedo effect does not dominate. IPSS together with a COTS magnetometer can achieve an attitude determination accuracy of 0.3–0.6${}^{\circ }$ depending on the orbit type and season. Since the albedo effect introduces mainly slowly varying system error, the estimated angular rate is quite accurate. The overall simulation results are consistent with the results of error analyses in Section 3.

### 4.3. On-Orbit Verification of IPSS

**Figure 18:**
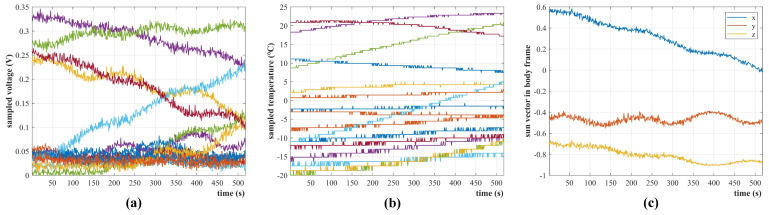
Raw measurements of IPSS extracted from the real−time telemetry data from 25 March 2021 03:04:45 UTC to 03:13:22 UTC. (**a**) sampled voltage curves of the 16 solar cells. (**b**) sampled temperature curves of the 16 solar cells. (**c**) the inversed sun vector in the satellite body frame by Q-SAT.

Q-SAT was launched atop the CZ-2D rocket on 6 August 2020, and has been working well for more than two years. Figure 18 gives the collected telemetry data when Q-SAT passed Changsha ground station from 25 March 2021 03:04:45 UTC to 03:13:22 UTC. The maximum voltage of each solar cell is about 0.34V, and the sampling fluctuation is about 5mV which is consistent with the experimental data shown in Figure 4 and Figure 5. The sampled temperature of the solar cell ranges from −25 ${}^{\circ }$C to 25 ${}^{\circ }$C. The temperature is lower than that in Figure 16 due to the extremely cold background of the universe (about 3 Kelvin). Figure 18c shows the inversed sun vector expressed in the satellite body frame calculated in real-time by Q-SAT.

**Figure 19:**
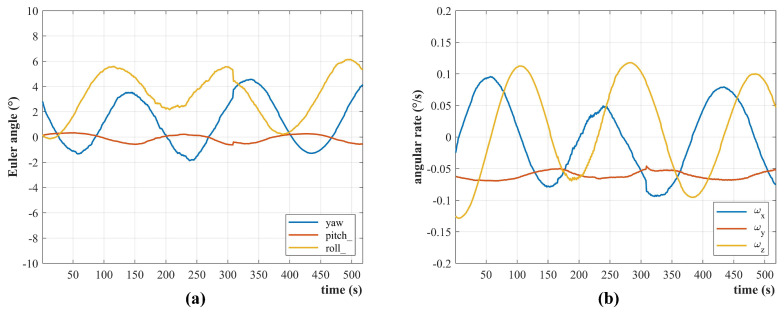
The on−orbit attitude determination results are calculated in real-time by Q-SAT. The data starts from 25 March 2021 03:04:45 UTC to 03:13:22 UTC. (**a**) The attitude is represented by Euler angles (3-2-1) with respect to the orbital coordination frame. (**b**) The angular velocities are defined with respect to the inertial system. The estimated angular velocity in the satellite body Y axis is close to the orbital angular velocity of Q-SAT.

The IPSS together with a COTS magnetometer, a bias momentum wheel, and three magnetorquers compose the sensors and actuators of the ADC system. Their parameters are the same as that listed in Table 3. Figure 19 presents the Euler angles (a) and angular velocities (b) estimated in real time by Q-SAT. The Euler angles are defined with respect to the orbital coordination system, while the angular velocities are defined with respect to the inertial system. As shown in Figure 19a, the pitch angle never exceeds 0.5${}^{\circ }$. The roll and yaw angle exhibit a phenomenon of precession with an amplitude of about 3${}^{\circ }$ due to the gyroscopic effect of the bias momentum wheel and the small control torque of the magnetorquers (about 1.5 × 10${}^{-4}$ N·m). The gyroscopic motion does not mean that the attitude determination error has the same magnitude, but the actual attitude of Q-SAT is determined by the intrinsic characteristics of the control framework. The estimated angular velocity in the satellite body Y axis is averaged to be −0.61${}^{\circ }$/s, which is closed to the orbital angular velocity of Q-SAT (−0.67${}^{\circ }$/s). Although Q-SAT is not equipped with a high-precision attitude determination device such as a star sensor for comparison, the smoothness of the estimated Euler angles and the consistency of the estimated angular velocity in the body Y axis have verified the on-orbit performance of the proposed IPSS.

## 5. Conclusions

This paper has mainly discussed the design, modeling, error analyses, and on-orbit verification of the integrated panoramic sun sensor (IPSS) atop the small spherical satellite Q-SAT. IPSS is essentially 16 temperature-compensated solar cells mounted on the surface of Q-SAT, which has maintained the spherical structure of the satellite for the most part. IPSS is designed to be fault tolerant and has full spherical coverage of 4π. IPSS also has negligible power consumption compared with more accurate digital sun sensors and star trackers, which is crucial for satellites with limited power budgets. The mechatronic design and mathematical model of the proposed IPSS are presented. In-depth error analyses and ground experiments are carried out to validate the overall design. IPSS can provide a sun vector estimation accuracy better than 1.5${}^{\circ }$ when the albedo effect does not dominate. Simulation results have also suggested that IPSS together with a COTS magnetometer can achieve an attitude determination accuracy of 0.3-0.6${}^{\circ }$ and 0.01${}^{\circ }$/s in various orbits and seasons. Although IPSS is designed for the spherical Q-SAT, it can also be used in other small satellites that have limited power budgets and do not require very high attitude determination accuracy.

## Figures and Tables

**Table 1:** Q-SAT Specifications.

COSPAR ID	2020-054B
diameter	510 mm
weight	23 kg
payload	dual frequency GNSS receiver
separation system	electromagnetic separation system
perigee	488.0 km
apogee	513.9 km
inclination angle	97.5${}^{\circ }$
orbit period	84.5 min
semi-major axis	6871 km

**Table 2:** Summary of factors that affect the on-orbit performance of IPSS.

Category	Factor	Parameter	Magnitude	Introduced Error
sampling error	current/voltage sampling error of solar cells	I/V	2.5 mA/5 mV	1.04${}^{\circ }$
	temperature sampling error	*T*	<3 ${}^{\circ }$C	<0.64${}^{\circ }$
manufacturing and installation	installation matrix error of solar cells	${\vec{n}}_{i}$	0.5${}^{\circ }$	0.16${}^{\circ }$
parameter error	resistance error of current sampling resistor	$R_{p}$	0.5%	0.14${}^{\circ }$
	temperature compensation coefficient error	*K*	10%	<0.50${}^{\circ }$
	error in max. generated current at $T_{0}$	$I_{\max,T_{0}}$	2 mA	0.48${}^{\circ }$
albedo and seasonal variations	Earth albedo effect	*E*	up to 40%	depends
	seasonal variations in sunlight intensity	*E*	3.4%	negligible

**Table 3:** Summary of the core parameters used in the simulations.

Parameter	Value
satellite weight	23 kg
satellite inertial matrix	$I_{xx}$ = 0.6349 kg · m${}^{2}$
	$I_{yy}$ = 0.7960 kg · m${}^{2}$
	$I_{zz}$ = 0.6238 kg · m${}^{2}$
	$I_{xy}$ = 0.0023 kg · m${}^{2}$
	$I_{yz}$ = 0.0019 kg · m${}^{2}$
	$I_{zx}$ = −0.0086 kg · m${}^{2}$
inertial matrix error of attitude filter	10%
magnetometer measurement error	250 nT
magnetic momentum of magnetorquer	3.4 A · m${}^{2}$
inertial of the bias momentum wheel	1.067 × 10${}^{-4}$ kg · m${}^{2}$
rotational speed of bias momentum wheel	2000.0 rpm
control frequency	1 Hz

**Table 4:** Summary of the performance of the proposed IPSS in simulation.

Season/Time of the Year	Local Time of Descending	Average Accuracy of IPSS	Attitude Determination Accuracy	
			Angle	Angular Rate
Spring Equinox	18:00	1.62${}^{\circ }$	0.32${}^{\circ }$	0.0014${}^{\circ }$/s
	12:00	3.12${}^{\circ }$	0.43${}^{\circ }$	0.0007${}^{\circ }$/s
Summer Solstice	18:00	2.30${}^{\circ }$	0.38${}^{\circ }$	0.0013${}^{\circ }$/s
	12:00	3.24${}^{\circ }$	0.61${}^{\circ }$	0.0013${}^{\circ }$/s

## Data Availability

The data presented in this study are available on request from the corresponding author. The data are not publicly available due to intellectual property protection.

## Abbreviations

The following abbreviations are used in this manuscript:

IPSS	Integrated Panoramic Sun Sensor
GNSS	Global Navigation Satellite System
ADC	Attitude Determination and Control
FOV	Field of View
CCD	Charge Coupied Device
A/D	Analog to Digital
CNC	Computerised Numerical Control Machine
COTS	commercial-off-the-shelf
IGRF	International Geomagnetic Reference Frame
TOMS-EP	Total Ozone Mapping Spectrometer Earth Probe
